# *BDNF*: mRNA expression in urine cells of patients with chronic kidney disease and its role in kidney function

**DOI:** 10.1111/jcmm.13762

**Published:** 2018-08-21

**Authors:** Nicole Endlich, Tim Lange, Jana Kuhn, Paul Klemm, Ahmed M. Kotb, Florian Siegerist, Frances Kindt, Maja T. Lindenmeyer, Clemens D. Cohen, Andreas W. Kuss, Neetika Nath, Rainer Rettig, Uwe Lendeckel, Uwe Zimmermann, Kerstin Amann, Sylvia Stracke, Karlhans Endlich

**Affiliations:** ^1^ Department of Anatomy and Cell Biology University Medicine Greifswald Greifswald Germany; ^2^ Clinic for Diabetes and Metabolic Diseases Karlsburg Hospital Dr. Guth GmbH & Co KG Karlsburg Germany; ^3^ Nephrological Center Medical Clinic and Policlinic IV University of Munich Munich Germany; ^4^ Department of Functional Genomics University Medicine Greifswald Greifswald Germany; ^5^ Institute of Bioinformatics University of Greifswald Greifswald Germany; ^6^ Department of Physiology University of Greifswald Karlsburg Germany; ^7^ Department of Medical Biochemistry and Molecular Biology University Medicine Greifswald Greifswald Germany; ^8^ Department of Urology University Medicine Greifswald Greifswald Germany; ^9^ Department of Pathology University of Erlangen‐Nürnberg Erlangen Germany; ^10^ Department of Internal Medicine A University Medicine Greifswald Greifswald Germany

**Keywords:** *BDNF*, biomarker, CKD, diabetes, podocyte

## Abstract

Podocyte loss and changes to the complex morphology are major causes of chronic kidney disease (CKD). As the incidence is continuously increasing over the last decades without sufficient treatment, it is important to find predicting biomarkers. Therefore, we measured urinary mRNA levels of podocyte genes *NPHS1, NPHS2, PODXL* and *BDNF, KIM‐1, CTSL* by qRT‐PCR of 120 CKD patients. We showed a strong correlation between BDNF and the kidney injury marker *KIM‐1*, which were also correlated with *NPHS1*, suggesting podocytes as a contributing source. In human biopsies, *BDNF* was localized in the cell body and major processes of podocytes. In glomeruli of diabetic nephropathy patients, we found a strong *BDNF* signal in the remaining podocytes. An inhibition of the *BDNF* receptor TrkB resulted in enhanced podocyte dedifferentiation. The knockdown of the orthologue resulted in pericardial oedema formation and lowered viability of zebrafish larvae. We found an enlarged Bowman's space, dilated glomerular capillaries, podocyte loss and an impaired glomerular filtration. We demonstrated that *BDNF* is essential for glomerular development, morphology and function and the expression of *BDNF* and *KIM‐1* is highly correlated in urine cells of CKD patients. Therefore, *BDNF* mRNA in urine cells could serve as a potential CKD biomarker.

## 1 INTRODUCTION

The “Greifswald Approach to Individualized Medicine (GANI_MED)” aims at the development of individualized diagnosis, prevention and therapy strategies for common diseases.^1^, ^2^ Therefore, patient cohorts were recruited and investigated under standardized and routine conditions. The cohort investigated in this report consisted of patients diagnosed with chronic kidney disease (CKD).^3^

CKD mostly affects risk groups like patients suffering from diabetes mellitus or hypertension.^4^, ^5^, ^6^ The disease is characterized by a decrease and subsequently by a loss of kidney function named end‐stage renal disease. Loss of renal function can only be compensated by renal replacement therapies like haemodialysis or transplantation. Until today, CKD is not reversible and it is therefore important to identify predictive biomarkers and possible target molecules allowing early detection and prevention.

As it became obvious that podocytes are associated with the development of CKD, this specific cell type was brought into the focus of research.^7^, ^8^ Podocytes, a post‐mitotic cell type, maintain the glomerular filtration barrier by their unique cellular structure, which includes major processes and foot processes covering the glomerular basement membrane (GBM) in a zipper‐like fashion.^9^, ^10^ Changes in these structures lead to an impairment of glomerular function and are related to several kidney diseases like focal segmental glomerulosclerosis (FSGS), minimal change disease (MCD) and diabetic nephropathy (DN).

As podocytes share certain structural and molecular biological characteristics with neurons, proteins involved in neuronal structural and physiological maintenance^11^, ^12^ are of great interest for podocyte research and might play a potential role as biomarkers. One of those neuron‐specific proteins is *brain‐derived neurotrophic factor* (*BDNF*), a neurotrophic factor which is involved in neurogenesis, neuronal survival,^13^, ^14^ branching^15^, ^16^ and synaptic growth.^17^ Thus, Ernfors et al^18^ have already shown that *BDNF* plays a key role in neuronal development, because heterozygous knockout (KO) mice showed decreased neuronal development and homozygous KO mice often die directly after birth. It has already been shown that *BDNF* binds to 2 different receptors—TrkB and p75, that are involved in cell survival and differentiation processes.^19^, ^20^ Recently, it has been reported that *BDNF* and TrkB are expressed in podocytes *in vivo*, being essential for actin polymerization and cell survival.^21^ As the actin cytoskeleton plays an important role for podocyte morphology and adhesion *in vivo*, and podocyte detachment is a major event in glomerulopathies, we investigated the expression of *BDNF* in cells appearing in the urine of patients suffering from CKD in an attempt to find out whether *BDNF* could be a suitable marker for the detection of DN.

As a second potential biomarker for glomerulosclerosis, we chose Hepatitis A virus cellular receptor 1 (*HAVCR1*) or kidney injury molecule‐1 (*KIM‐1*), a transmembrane protein that is not or at very low levels expressed in healthy kidneys.^22^, ^23^, ^24^, ^25^ Interestingly, Zhao et al^26^ found *KIM‐1* being up‐regulated in parietal epithelial cells and dedifferentiated podocytes of diabetic rats. Furthermore, recent findings show that the expression of *KIM‐1* reduces the negative effects of acute kidney injury by inducing phagocytosis.^27^ Therefore, we selected *KIM‐1* to proof whether it could be used as a potential glomerular biomarker.

To study the influence of *BDNF* on podocyte development and glomerular morphology *in vivo*, we took larval zebrafish as a well‐established model organism. The zebrafish larva is ideal for podocyte research^28^, ^29^, ^30^ as it develops a functioning glomerulus during 48‐56 hours post‐fertilization (hpf),^31^, ^32^ which can be studied directly in living larvae by 2‐photon microscopy (2‐PM).^28^, ^33^, ^34^ Moreover, by the use of the morpholino technology, specific proteins can easily be knocked down.

Zebrafish express a *bdnf* orthologue, whose amino acid sequence is 91% identical to human *BDNF*.^35^ Although the *BDNF* sequence is rather conserved among these species, little is known about the function of *bdnf* in the zebrafish pronephros. A recent study has shown that there is a beneficial, microRNA‐mediated effect on actin polymerization in adriamycin‐induced podocyte damage emphasizing the important role for *BDNF* in kidney homoeostasis.^21^

Our study shows that the mRNA expressions of *BDNF*, a newly identified podocyte gene, and of *KIM‐1*, an injury‐induced protein, are highly correlated in urine cells of CKD patients and secondly that the expression is associated with DN. Moreover, we show the importance of *BDNF* for glomerular function in zebrafish larvae and in isolated murine glomeruli.

## 2 METHODS

### 2.1 Study participants

Participants were recruited in the GANI_MED nephrology cohort.^3^ All participants signed informed written consent forms. The study is consistent with the principles of the declaration of Helsinki, as reflected by an a priori approval of the Ethics Committee of the University of Greifswald.

### 2.2 Clinical sample collection

A total of 120 urine samples were collected from participants who had known CKD with or without hypertension and/or diabetes. We used 50‐100 mL morning urine. Only in 5% of cases, the urine volume was <50 mL. Unfortunately, the HbA1c value of 1 patient was not available. The time period from urine voiding until processing never exceeded 4 hours, as we found out that in this time viable cells could still be cultivated.

### 2.3 Urine processing

Urine was centrifuged in a 50 mL centrifuge tube at room temperature (RT) for 3 minutes at 2100 *g*. The urine pellet was resuspended in 1 mL phosphate‐buffered saline (PBS), transferred to a 1.5 mL centrifuge tube and then centrifuged at 12 000 *g* for 1 minute at RT. The supernatant was discarded. The washed urine pellet was resuspended in 900 μL Phenol/Guanidine‐based Qiazol lysis reagent (Qiagen, Hilden, Germany) and then short‐term stored at −20°C until use.

### 2.4 Kidney specimens

Kidney tissue for immunofluorescence was obtained by percutaneous renal biopsy from patients undergoing diagnostic evaluation. Biopsies from 2 subjects with diagnosed DN were investigated. The histopathological diagnosis included the following: diabetic glomerulosclerosis (patient 1) and FSGS with tubular changes (patient 2). Control kidney tissue was taken from normal kidney parts of a renal tumour surgery patient. The clinical‐functional diagnosis included the following: slight restriction in GFR and arterial hypertrophy. An informed consent was obtained from the donor.

### 2.5 Podocyte de‐/differentiation assay

All animal experiments were performed in accordance with national animal protection guidelines that conform to the National Institutes of Health Guide for the Care and Use of Laboratory Animals and were approved by the local governmental authorities. The podocyte dedifferentiation assay was performed as described by Kindt et al.^36^ Glomeruli were treated with ANA‐12 (1‐100 μmol/L, Sigma‐Aldrich). After 6 days, the cyan fluorescent protein (CFP) intensity was quantified. Therefore, *z*‐stacks of 50 to 80 glomeruli were recorded with the aforementioned confocal laser scanning microscope. Mean fluorescence intensity per glomerulus was calculated after background correction. Half maximal inhibitory concentrations (IC_50_) were calculated by fitting the data to a sigmoidal dose‐response regression curve using Prism 5.01 (GraphPad Software, San Diego, CA, USA). RNA sequencing was performed as previously described.^37^

### 2.6 Zebrafish strains

The following zebrafish strains were used: ET (Tg(*wt1a*:eGFP); mitfa^w2/w2^; roy^a9/a9^), CADE (Tg(*fabp10a*:DBP‐eGFP); mitfa^w2/w2^; roy^a9/a9^).^38^ All zebrafish strains were raised, mated and maintained in E3 medium at 28.5°C, as previously described.^30^, ^39^

### 2.7 Morpholinos injection

Translation‐blocking bdnf morpholinos (bdnfMO) were manufactured by Gene Tools LCC (Philomath, OR, USA). As negative control, we used standard control morpholinos (CtrlMO) offered by Gene Tools. The morpholinos were diluted to 1 mmol/L. A volume of approximately 3 nL per zebrafish was injected into 2 to 4‐cell stage fertilized eggs using a microinjector (Transjector 5246, Eppendorf, Hamburg, Germany).

### 2.8 Immunohistology

Immunohistology for cryosections was performed as described previously.^39^, ^40^

### 2.9 Zebrafish in vivo microscopy

In vivo imaging was performed as previously described.^28^, ^34^, ^40^

### 2.10 Statistical analysis

Urine expression data were log‐transformed for all correlation analyses. Associations between potential biomarkers were assessed using Pearson correlation followed by the Benjamini‐Hochberg procedure. Comparisons between groups were performed as indicated. All comparisons between 2 groups concerning zebrafish experiments were done with the Mann‐Whitney *U* test. All statistical analyses were performed using LABMAT version 2013 and SPSS V. 21.

## 3 RESULTS

### 3.1 Baseline characteristics of patients

To identify individual prognostic biomarkers for CKD, we analysed a panel of potential urinary biomarkers in 120 GANI_MED renal study participants. The baseline characteristics are shown in Table 1. The investigated patient group consisted of 45 women (37.5%) and 75 men (62.5%) with a mean age of 64.3 years. All patients were afflicted with CKD. The group included 33 diabetes patients and 75 dialysis patients. Mean estimated glomerular filtration rate (eGFR) was 23.2 mL/min/1.73 m^2^, and 110 patients had an eGFR less than 60 mL/min/1.73 m^2^. The mean urinary albumin‐to‐creatinine ratio (uACR) was 1245 mg/g. The diabetic group consisted of 19 male and 14 female patients, whereas the non‐diabetics included 55 men and 31 women. For one study participant, there was no information about the diabetic status. The mean age was 65.9 years in diabetics and 63.6 years in non‐diabetics. The diabetic patients had a mean eGFR of 23.9 mL/min/1.73 m^2^ compared to 22.7 mL/min/1.73 m^2^ in non‐diabetic patients. This difference was not statistically significant. There was also no statistically significant difference concerning the uACR, which was 1531 mg/g in diabetics and 1149 mg/g in non‐diabetics. The group of diabetics included 32 patients with an eGFR lower than 60 mL/min/1.73 m^2^ and 16 dialysis patients. The group of non‐diabetics included 77 patients with an eGFR lower than 60 mL/ min/ 1.73 m^2^ and 59 dialysis patients. Again, these differences were not statistically significant.

**Table 1:** Patient characteristics

Variables	Total	Diabetics	Non‐diabetics
n	120	33	86
Sex (m/f)	75/45	19/14	55/31
Mean age (y)	64.3 ± 15.7	65.9 ± 13.3	63.6 ± 16.5
Mean eGFR mL/min/1.73 m^2^	23.2 ± 20.9	23.9 ± 16.1	22.7 ± 22.4
eGFR<60 mL/min/1.73 m^2^	110/120	32/33	77/86
Mean UACR (mg/g)	1245 ± 2199	1531 ± 2654	1149 ± 1994
Dialysis	75/120	16/33	59/86

m = male; f = female; eGFR = estimated glomerular filtration rate, UACR = urinary creatinine‐albumin ratio.

### 3.2 Correlations between urine mRNA levels

**Figure 1:**
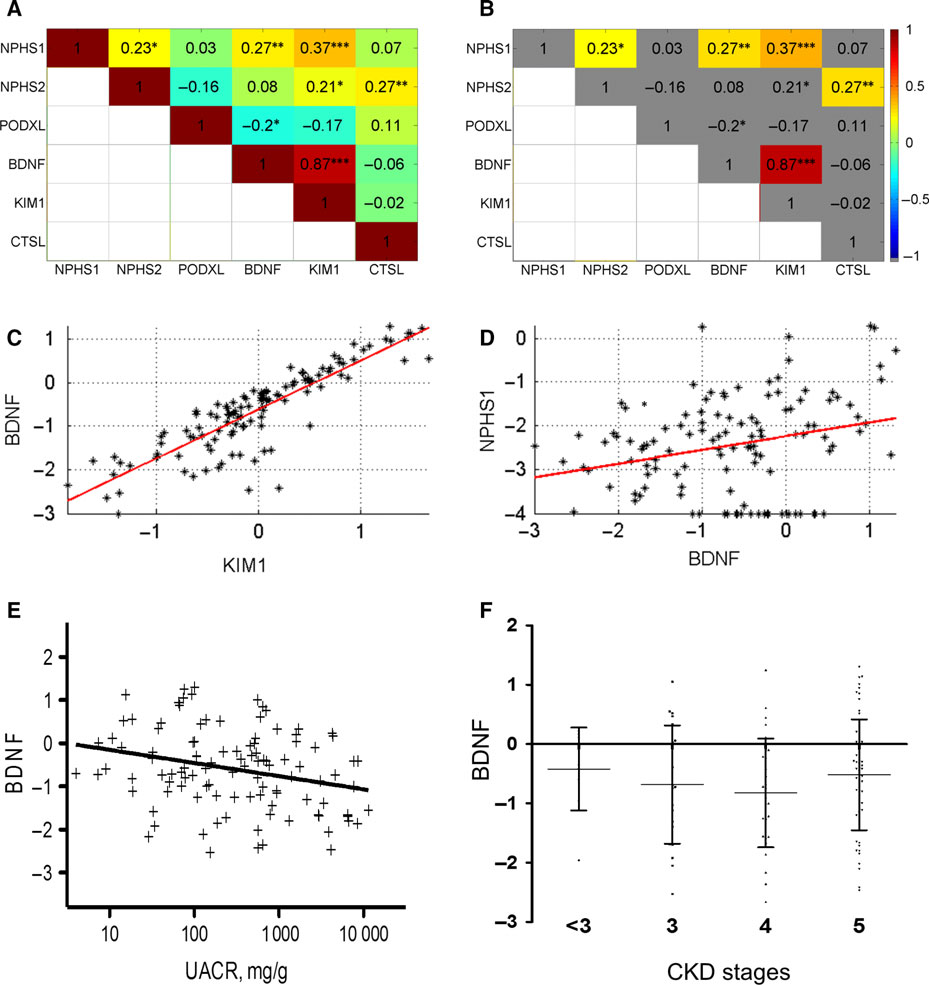
Pearson correlation of urine sediment mRNA expression. mRNA expression was determined by qRT‐PCR and normalized to *GAPDH* and the interrun calibrator. Pearson correlations are shown in the coloured boxes (A). Pearson correlations after Benjamini‐Hochberg procedure are shown in B, where grey boxes indicate non‐significant correlations and coloured boxes indicate significant values. *BDNF* mRNA was significantly correlated with mRNA of *KIM‐1* and *NPHS1* as also shown in C and D. *KIM‐1* mRNA is significantly correlated with *NPHS1* as well. *BDNF* mRNA is correlated with uACR (E). There is no significant difference in BDNF mRNA expression between different CKD stages [stage <3: n = 9, stage 3: n = 21, stage 4: n = 31, stage 5: n = 58]; (F). [*P < .05; **P < .01; ***P < .001]

To investigate whether the mRNA levels were interrelated, we performed a correlation analysis (Figure 1A) followed by the Benjamini‐Hochberg procedure to determine statistical significance (Figure 1B). We found a strong positive correlation between the expressions of *BDNF* and *KIM‐1* (*R* = 0.87, *P* = 2.3 × 10^−38^, Figure 1C). Interestingly, we also observed significant correlations between the expressions of *BDNF* and the podocyte marker *NPHS1* (*R* = 0.27, *P* = .0025, Figure 1D) as well as between the expressions of *KIM‐1* and *NPHS1* (*R* = 0.37, *P* = 3.7 × 10^−5^). These findings suggest that podocytes might contribute to the population of *BDNF*‐ and *KIM‐1*‐expressing cells that are detectable in the urine. Additionally, there were statistically significant correlations of the podocyte marker *NPHS2* with *NPHS1* (*R* = 0.23, *P* = .011) as well as with *CTSL‐1* (*R* = 0.27, *P* = .0026). Other significant correlations were not detected.

### 3.3 Correlation analysis between urine mRNA levels and clinical parameters revealed a negative correlation between *BDNF* and uACR

To identify possible correlations between the investigated expressions of urinary cell pellet mRNA species and clinical parameters, we applied the Pearson correlation analysis corrected by the Benjamini‐Hochberg procedure for the expression of the 3 podocyte markers *NPHS1*,* NPHS2*,* PODXL*, the 3 biomarker candidates *BDNF*,* KIM‐1* and *CTSL‐1* and the clinical parameters eGFR, HbA1c and uACR. These calculations revealed a significant negative correlation between *BDNF* and uACR (*P* = .0046; Figure 1E). *BDNF* and eGFR were not correlated with each other. Additionally, there was no significant difference in BDNF expression between patients of different CKD stages (Figure 1F). Furthermore, we did not find any significant correlation between any of the other parameters in the observed patient group (data not shown).

### 3.4 Sex‐specific expression of *BDNF* and *KIM‐1* in diabetic and non‐diabetic patients

As our cohort shows a typical frequency distribution of nephropathies with the largest proportion suffering from diabetes, which is one of the major causes of CKD, we investigated the expression of *BDNF* and *KIM‐1* in diabetic and non‐diabetic patients (Figure S1). We observed that the expressions of *BDNF* and *KIM‐1* were significantly higher in diabetic than in non‐diabetic patients (*P* = .04 and *P* = .02, respectively). The mean *BDNF* expression in diabetics reached a value of 2.07, whereas non‐diabetics showed a lower expression value of 1.30. Similar results were found for *KIM‐1*. The expression of *KIM‐1* was almost twice as high in diabetic patients than in non‐diabetic patients (5.79 vs 2.97).

Interestingly, we found that the expressions of *BDNF* and *KIM‐1* were almost twice as high in females than in males (Figure S1), indicating a sex‐dependent expression of *BDNF* and *KIM‐1*. Thus, in females suffering from DN, the expressions of *BDNF* and *KIM‐1* were 3.97 and 8.07, respectively, compared to 2.15 and 4.75, respectively, in non‐diabetic females. The associations of *BNDF* and *KIM‐1* with DN were not detected in males (Figure S1). There was a statistically significant difference in *BDNF* mRNA expression between female and male diabetics but not between female and male non‐diabetics. In contrast, *KIM‐1* mRNA expression did not significantly differ between female and male diabetics or non‐diabetics.

### 3.5 *BDNF* and *KIM‐1* expressions are up‐regulated in podocytes of patients with DN

**Figure 2:**
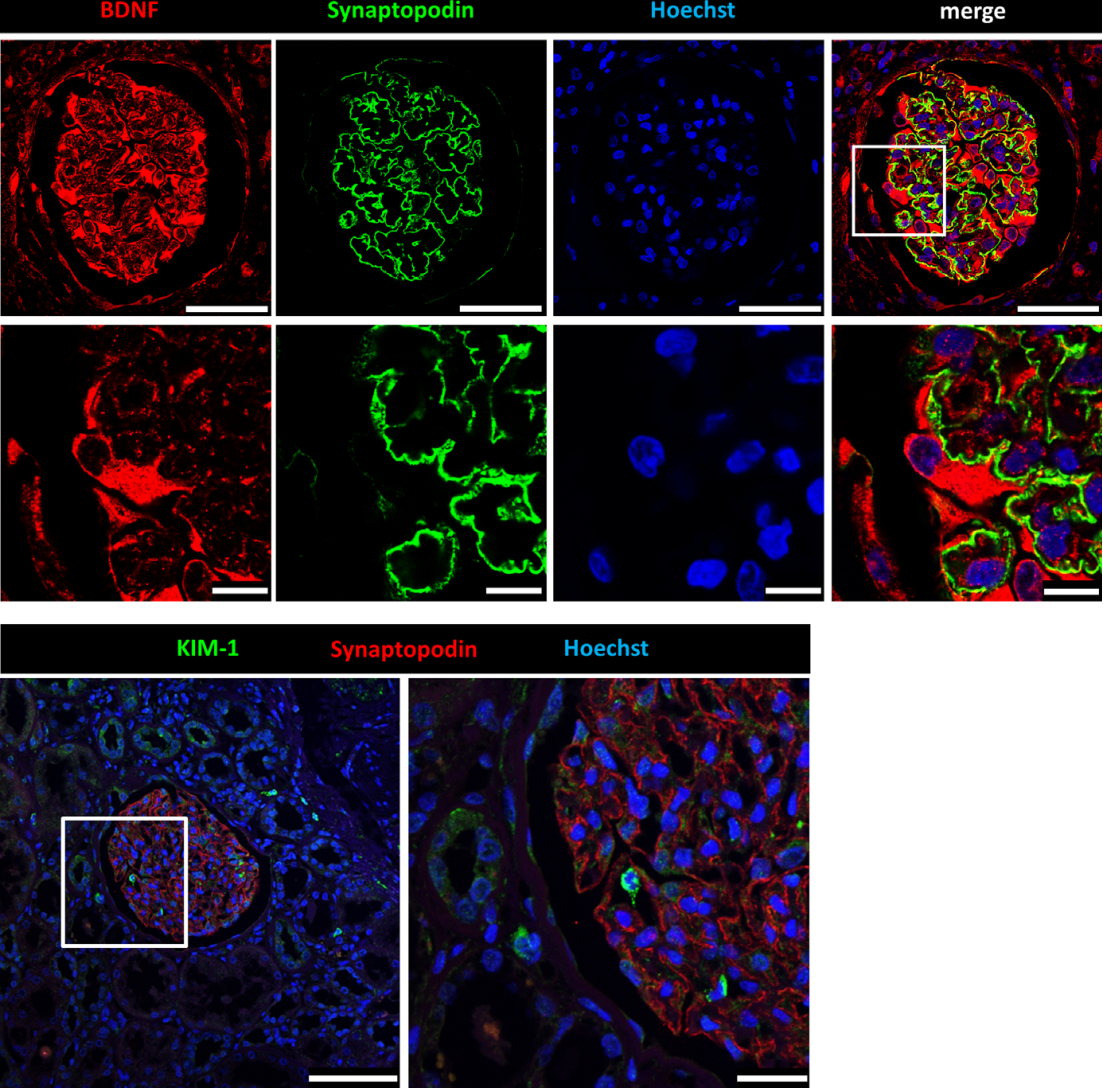
Immunofluorescence staining of healthy human kidney sections for *BDNF* and *KIM‐1*. Normal formalin‐fixed paraffin embedded kidney sections were stained for *BDNF* (red), the podocyte marker protein synaptopodin (green) and nuclei (blue) by Hoechst. *BDNF* was mainly expressed in the cell body and major processes of podocytes. There was moderate expression of synaptopodin in parietal epithelial cells. [Scale bars upper panel = 50 μm. Scale bars lower panel = 10 μm] Kidney sections were also stained for *KIM‐1* (green), synaptopodin (red) and nuclei (blue) by Hoechst. Very few *KIM‐1* positive cells were visible within the glomerulus. Only a few non‐glomerular cells were stained. [Scale bar left picture = 100 μm. Scale bar right picture = 25 μm]

**Figure 3:**
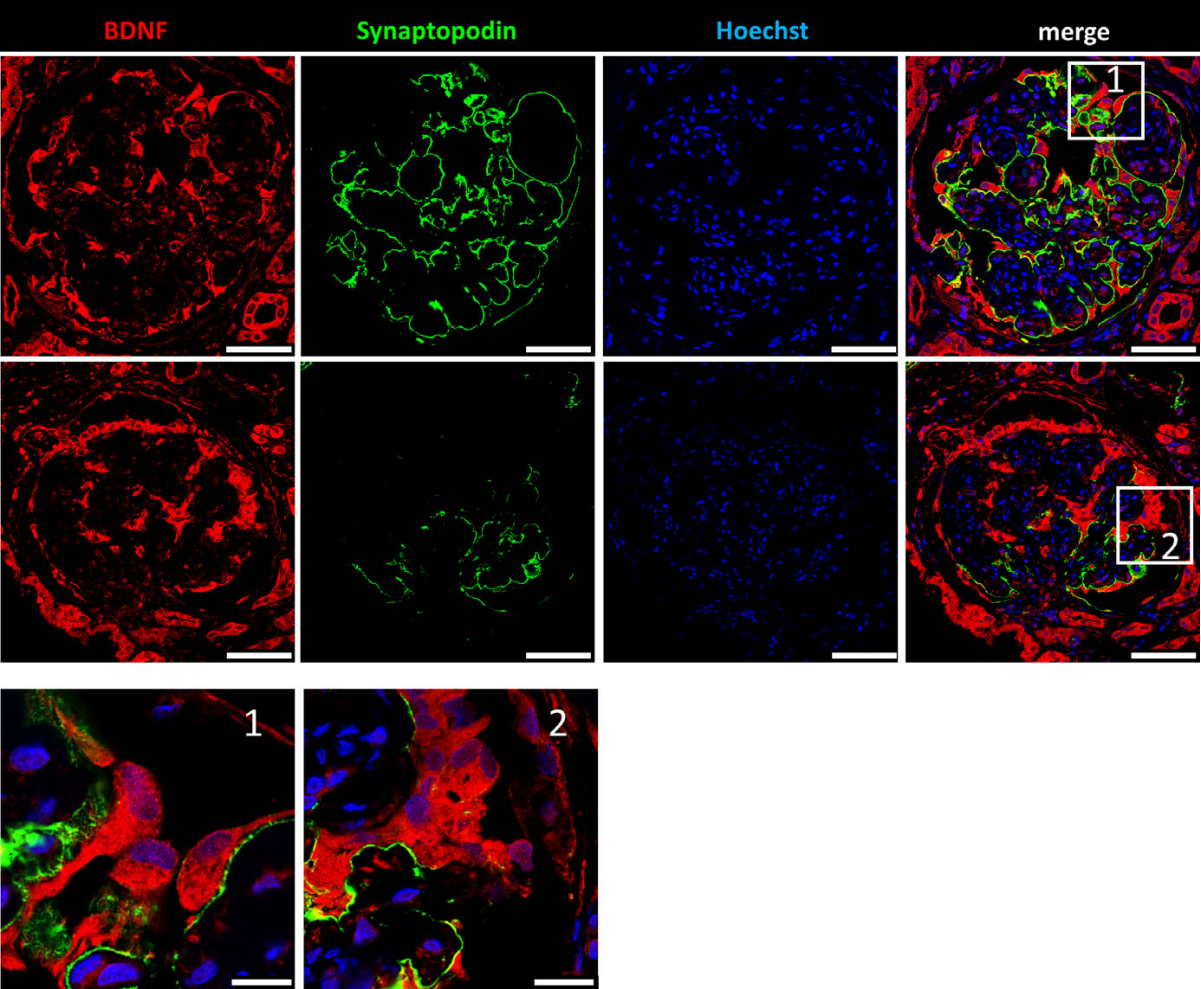
Immunofluorescence staining of human diabetic nephropathy (DN) kidney sections for *BDNF*. Formalin‐fixed paraffin embedded DN kidney sections were stained for *BDNF* (red), synaptopodin (green) and nuclei (blue) by Hoechst. Fewer *BDNF*‐expressing cells were found in glomeruli of DN kidney sections than in healthy kidneys from Figure 2. Cells still expressing the podocyte marker protein synaptopodin show an enhanced *BDNF* intensity (magnification 1 and 2). [Scale bars upper panels = 50 μm. Scale bars 1/2 = 10 μm]

**Figure 4:**
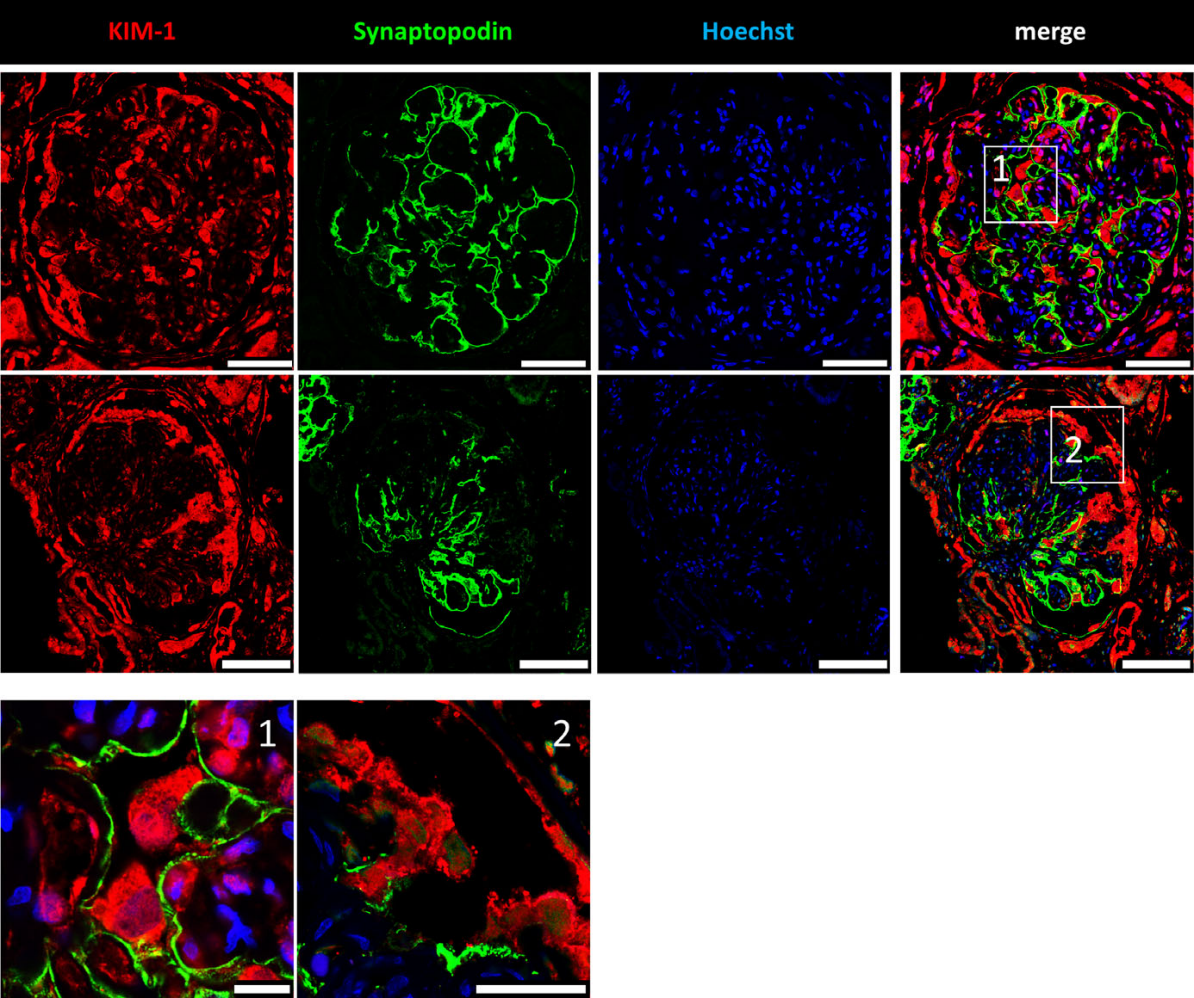
Immunofluorescence staining of human diabetic nephropathy (DN) kidney sections for *KIM‐1*. Formalin‐fixed paraffin embedded DN kidney sections were stained for *KIM‐1* (red), synaptopodin (green) and nuclei (blue) by Hoechst. A significant increase in the *KIM‐1* expression was found in podocytes as well as in other epithelial cells. Higher magnifications are shown in 1 and 2. [Scale bars upper panel = 50 μm. Scale bars lower panel = 75 μm. Scale bars 1/2 = 10 μm]

As described previously, *BDNF* is expressed in a range of different tissues and cell types. On the basis of our finding that the urine mRNA levels of *BDNF* and the podocyte‐specific marker *NPHS1* were statistically associated with each other, we investigated the expression of *BDNF* in human glomeruli by immunofluorescence staining. As shown in Figure 2, *BDNF* was expressed in glomeruli of normal human kidney sections, especially in podocytes and to some extent also in parietal epithelial cells. As the localization of synaptopodin was mainly restricted to podocyte foot processes and no co‐localization was found between synaptopodin and *BDNF* in podocytes, we conclude that *BDNF* is mainly localized in the major processes and in the cell body of podocytes. This was confirmed by co‐staining with the slit diaphragm protein nephrin (Figure S3). In renal biopsies from patients suffering from DN, we found an up‐regulation of the fluorescence intensity of the remaining podocytes as identified by co‐staining with synaptopodin (Figure 3). The subpodocyte space was lost in these biopsies. Confirming these findings, microarray analysis of glomeruli from patients suffering from DN showed a significant 2.0‐fold enhanced expression of *BDNF* and a 1.3‐fold enhanced expression of its receptor TrkB compared to glomeruli from control individuals. Moreover, we further confirmed the increase of BDNF expression (1.2‐fold) in protein‐overload experiments in cultured murine podocytes (Figure S4). In contrast to *BDNF*,* KIM‐1* was never observed in podocytes and rarely found in other cells of normal glomeruli (Figure 2). However, in patients with DN, a strong up‐regulation of *KIM‐1* in podocytes was detected (Figure 4). Additionally, sequencing data of glomeruli from our dedifferentiation assay^36^ revealed a 139‐fold increased Kim‐1 expression in 3‐day cultivated glomeruli compared to freshly isolated glomeruli.

### 3.6 *BDNF* stabilizes podocyte differentiation in cultured glomeruli

We recently established a novel assay to study podocyte de‐/differentiation in isolated glomeruli of mice expressing CFP under control of the *Nphs1* promoter.^36^ As podocytes of isolated glomeruli spontaneously dedifferentiate in cell culture accompanied by a decrease in *Nphs1* expression, we used the *Nphs1*‐dependent CFP expression to quantify podocyte dedifferentiation. Isolated glomeruli were incubated with various concentrations (1‐100 μmol/L) of the selective TrkB inhibitor ANA‐12. After 6 days, CFP intensity was decreased concentration‐dependently by ANA‐12 with an IC_50_ value of 19.6 μmol/L (Figure S2), indicating an involvement of *BDNF* in podocyte differentiation.

### 3.7 The knockdown of *BDNF* induces proteinuria in zebrafish larvae

**Figure 5:**
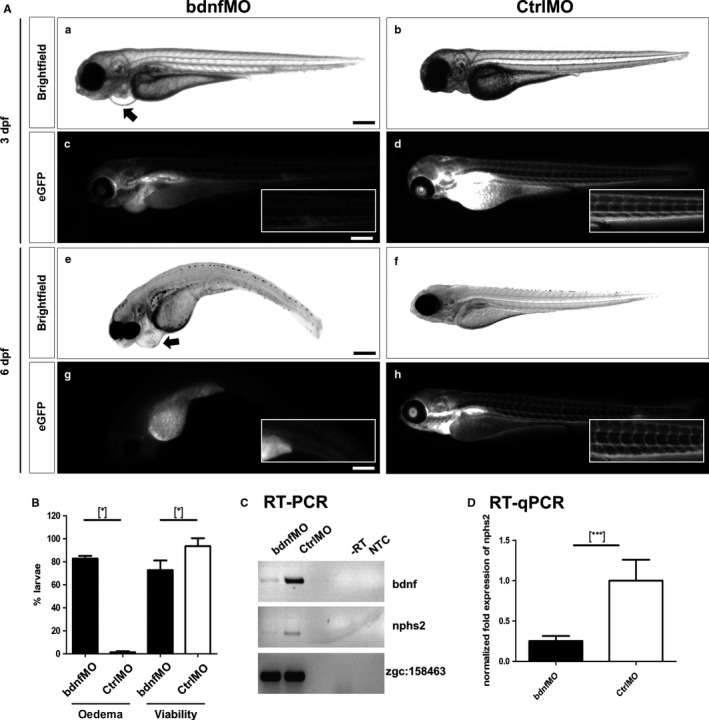
*Bdnf* knockdown in zebrafish larvae leads to pericardial oedema formation, impaired glomerular filtration and down‐regulation of podocyte‐specific genes. *Bdnf* morpholinos (bdnfMO) and control morpholinos (CtrlMO) were injected in *ET* zebrafish eggs. Brightfield pictures were taken 3 dpf (A; a and b) and 6 dpf (A; e and f). bdnfMO‐injected larvae exhibit a higher rate of pericardial oedema development and a lower viability than normal controls (B). Morpholino injection in *CADE* larvae reveals an impairment of glomerular filtration barrier function in bdnfMO‐injected larvae 3 dpf (A; c) and 6 dpf (A; g) compared to CtrlMO‐injected larvae (A; d and f). The knockdown of *bdnf* was verified by RT‐PCR (C). The down‐regulation of the podocyte marker *nphs2* was verified on the mRNA level by RT‐PCR (C) and qRT‐PCR (D). Expression levels were normalized to *zgc:158463* in RT‐PCR and to *zgc:158463* and *eef1a1/1* in qRT‐PCR by the ΔΔCt method. [Scale bars = 500 μm] [*P < .05, ***P < .001]

To study the function of *bdnf in vivo*, we performed a *bdnf* knockdown (KD) with specific morpholinos. To this end, we generated a translation‐blocking morpholino (bdnfMO). Three days after injection of the bdnfMO, 82.7 ± 2.0% of the zebrafish larvae had developed severe pericardial oedema (arrow in Figure 5A), a hallmark of impaired kidney function. In contrast, only 1.3 ± 0.8% of CtrlMO‐injected larvae developed pericardial oedema (Figure 5A,B). In addition, bdnfMO‐injected larvae were less viable (72.9 ± 7.1%) compared with larvae injected with CtrlMO (93.6 ± 6.0%) (Figure 5B). To investigate whether the pronephros of the zebrafish larvae was affected by the KD of *bdnf*, we used the transgenic zebrafish strain CADE expressing an eGFP‐tagged vitamin D‐binding protein in the blood which cannot pass the intact filtration barrier. In contrast to CtrlMO‐treated larvae, we observed a nearly complete loss of eGFP fluorescence in the blood of *bdnf* KD larvae at 3 and 6 dpf (Figure 5A), indicating leakage of the filtration barrier due to *bdnf* KD.

### 3.8 *BDNF* is important for the proper morphology of zebrafish glomeruli as well as for the expression of nephrin and podocin

**Figure 6:**
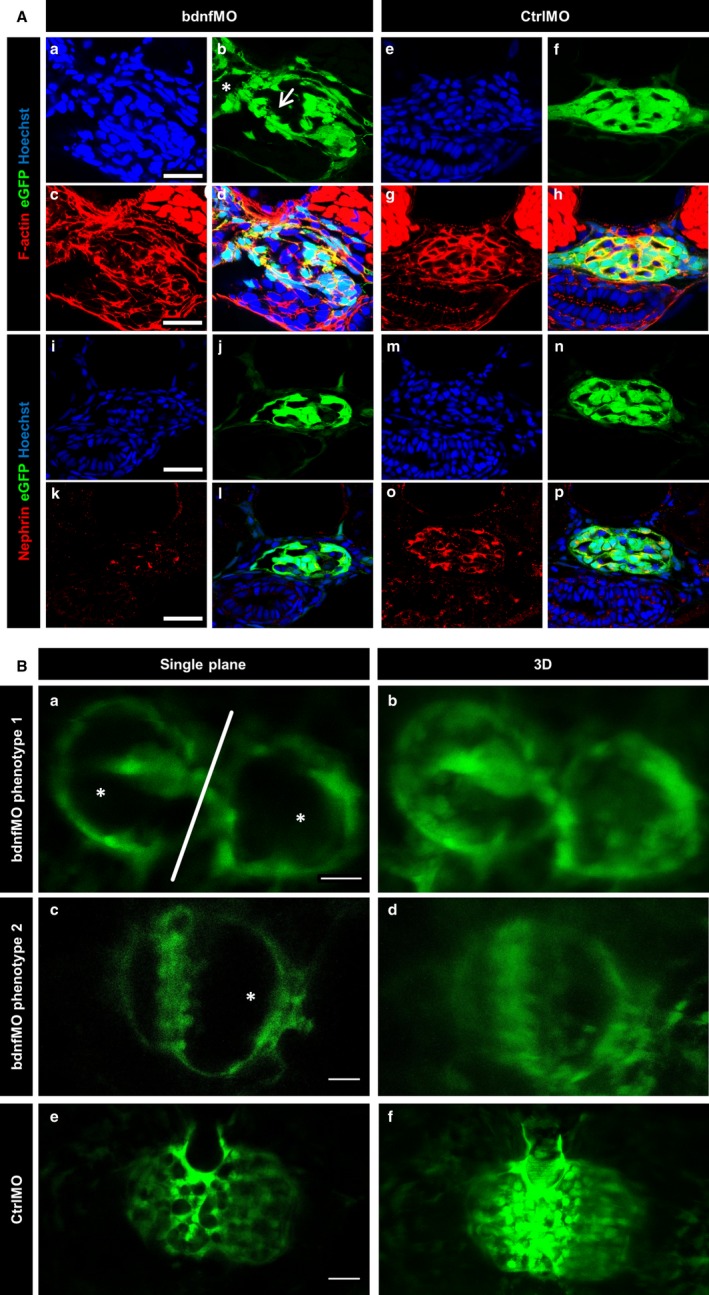
*Bdnf* knockdown in zebrafish larvae leads to morphological changes in the glomerulus and the down‐regulation of podocyte marker proteins. BdnfMO and CtrlMO were injected in ET eggs expressing eGFP specifically in podocytes. Cryosections were counterstained for F‐actin by phalloidine (red) and nuclei by Hoechst (blue) (A; a‐h). bdnfMO‐treated larvae show an enlarged glomerular tuft (A; b arrow) and Bowman's space (A; b asterisk) compared with the normal morphology of the CtrlMO‐treated larvae (A; e‐h). Counterstaining of the slit diaphragm protein nephrin (red) and nuclei by Hoechst (blue, A; i‐p) reveals a down‐regulation of nephrin due to bdnfMO treatment (A; k) compared to CtrlMO‐treated larvae (A; o). [Scale bars = 20 μm] *In vivo* microscopy reveals 2 different phenotypes of bdnfMO‐treated larvae. Phenotype 1 is characterized by unfused glomeruli (B; a white line and b), a reduced number of podocytes and a dilatation of Bowman's space and the glomerular tuft (B; a asterisks). The second phenotype is characterized by the absence of podocyte major processes, a reduced podocyte number and a dilatation of Bowman's space and the glomerular tuft (B; c asterisk and d). CtrlMO‐injected larvae show a normal glomerular morphology with well‐shaped major processes (B; e and f). [Scale bars = 20 μm]

The KD of *bdnf* was verified by RT‐PCR showing reduced intensities of the specific amplicon in bdnfMO‐injected larvae compared with CtrlMO‐injected larvae (Figure 5C). Furthermore, *bdnf* KD larvae showed reduced expression of *nphs2* (podocin) in RT‐PCR analysis (Figure 5C), which was confirmed by qRT‐PCR (Figure 5D). To study the glomerular morphology, we stained cryosections of zebrafish larvae, utilizing the ET strain that expresses eGFP specifically in podocytes.^28^, ^30^ After staining the F‐actin cytoskeleton with Alexa‐546 phalloidin, we observed significant changes in the morphology of the glomeruli in response to the KD of *bdnf*. In addition to an enlargement of the glomerular tuft and Bowman's space, we observed a reduced number of podocytes in *bdnf* KD larvae (3 dpf) in contrast to CtrlMO‐treated zebrafish larvae (Figure 6A). Moreover, immunohistological staining for nephrin revealed a significant reduction of the slit membrane protein due to the KD of *bdnf* in the zebrafish larvae (Figure 6A).

### 3.9 *In vivo* microscopy of *bdnf* knockdown larvae

Using *in vivo* 2‐photon microscopy (2‐PM) of *bdnf*MO‐injected *ET* larvae, we found 2 different phenotypes. Both phenotypes showed fewer podocytes and a dilated bowman's space (Figure 6Bc, asterisks) compared with controls (Figure 6Be,f). No major processes could be distinguished in *z*‐stacks of glomeruli of KD larvae (Movie S1), while control larvae showed a normal glomerular morphology and well‐shaped major processes (Movies S3 and S4). The second phenotype showed hindered fusion of the 2 glomeruli in the midline (Figure 6Ba, white line, Movie S2), indicating disturbance of the migration of the renal primordia (Figure 6A).

## 4 DISCUSSION

Biomarkers from non‐invasive sources like urine are of growing interest in CKD research, as they seem to be a promising diagnostic tool for early detection of the disease. Conventional parameters like proteinuria, cystatin C and creatinine are established markers of kidney function, which are positively correlated with kidney dysfunction.^41^, ^42^, ^43^, ^44^, ^45^ Because they are only measurable at a relatively progressed disease state, the establishment of markers for an earlier disease state is needed. In this study, we investigated the mRNA expression levels of potential predictive biomarkers for CKD in the urine sediment. Previous studies identified a variety of predictive biomarkers from urine sediment mRNA for non‐malignant kidney diseases.^46^, ^47^, ^48^, ^49^ In the present study, we found a strong and highly significant positive correlation between *BDNF* mRNA levels and *KIM‐1* mRNA levels. *KIM‐1*, which is endogenously expressed at a very low levels, is a well‐established urinary biomarker for acute kidney injury and is also known to be positively correlated with tubular and tubulointerstitial injury as well as with glomerular damage.^26^, ^50^, ^51^, ^52^ This is in a very good agreement with our findings, as we could also detect glomerular expression of *KIM‐1* by immunofluorescence staining and in murine glomeruli of our podocyte dedifferentiation assay. Therefore, *BDNF* mRNA levels might serve as a new potential biomarker for glomerular kidney injury. Interestingly, we observed a statistically significant up‐regulation of both mRNA species in diabetic patients compared with non‐diabetic patients and for *BDNF* in a sex‐specific manner. Sex specificity in kidney injury has been described earlier,^53^, ^54^ but seems to be relatively underestimated. *BDNF* mRNA levels were also negatively correlated with uACR, indicating a possible influence on glomerular filtration barrier function. The non‐correlation of urinary *BDNF* levels and eGFR may suggest that podocytes with high levels of BDNF possibly detach at a lower rate. Podocyte loss to a certain extent is a main event in glomerulopathies.^55^, ^56^ Accordingly, we also found a positive correlation of *KIM‐1* and *BDNF* with the podocyte marker *NPHS1*, suggesting that their expression might be, at least partially, podocyte‐derived. Currently, there is no known cellular mechanism directly linking *KIM‐1* and *BDNF*. Interestingly, recent studies have shown that both proteins are involved in autophagy, an important process for cell survival.^57^, ^58^, ^59^

Due to the findings described above, we further investigated the role of *BDNF in vitro* and *in vivo*. To date, only one study^21^ has addressed the role of *BDNF* in kidney function with a special focus on podocytes. Li and coworkers revealed a critical role for *BDNF* in podocyte cytoskeletal maintenance. They showed that *BDNF* TrkB‐dependently up‐regulates actin polymerization in podocytes through the regulation of the microRNA‐132/134 *in vitro*. Exogenous application of *BDNF* led to more developed stress fibres and ramifications of podocytes and could ameliorate podocyte damage by puromycin aminonucleoside, adriamycin and protamine sulphate. They showed that *BDNF*‐mediated TrkB activation has a stabilizing effect on podocyte homoeostasis as well as a rescuing effect in different podocyte injury models. These results suggest that the detection of *BDNF* in the urine of patients might be an indication of stressed podocytes that started to activate their “survival factor” *BDNF*.

As podocytes and neurons share some common features, it was predictable that *BDNF* may also be expressed in both cell types, as is the case for other proteins.^11^, ^12^ In keeping with this notion, we found that *BDNF* was expressed in the cell body and in major processes of podocytes in human kidney biopsies. Surprisingly, we did not see any co‐localization with synaptopodin, a podocyte‐specific protein which is exclusively expressed in podocyte foot processes, indicating that the localization of *BDNF* is restricted to the cell body and the major processes.

Looking at sectioned kidney biopsies of patients, which were diagnosed with DN, we observed less *BDNF*‐expressing podocytes, but podocytes that still expressed *BDNF* showed an increased expression, which could serve as a potential biomarker in the diagnostic evaluation of renal biopsies. These results were confirmed by microarray analyses of renal tissue from DN patients, where we have also detected an up‐regulation of the BDNF receptor TrkB, underlining the importance of *BDNF* for podocyte homoeostasis.

As podocyte dedifferentiation is a critical step in the progression of DN, we applied our well‐established dedifferentiation assay to study the influence of *BDNF* on podocyte differentiation.^36^ We could show that the inhibition of the TrkB receptor, which mediates for *BDNF* signalling, led to decreased nephrin promotor activity and therefore to increased podocyte dedifferentiation in murine glomeruli.

Only little is known about the effect of a *BDNF* KO on kidney homoeostasis *in vivo*. As *BDNF* KO mice die directly after birth and no kidney‐specific phenotypical impacts have been published,^18^ we selected the zebrafish larva as model organism. Zebrafish larvae are relatively easy to breed and show a glomerular morphology similar to that of mammals with one glomerulus connected to two tubules in their first functional state, the pronephros.^31^, ^32^ Another advantage is their applicability for *in vivo* microscopic techniques like 2‐PM which can track changes in morphology and function.^28^, ^34^ *bdnf* KD larvae developed pericardial oedema as an indicator for an impairment of the glomerular filtration barrier. This finding could be confirmed by a decrease in the intravascular eGFP intensity in bdnfMO‐treated *CADE* larvae, also indicating a leaky filtration barrier.^28^, ^30^ We also found a reduced expression of the podocyte markers podocin and nephrin, and a disrupted F‐actin structure in bdnfMO‐treated larvae, which are suggestive of podocyte loss.

A positive influence of *BDNF* on the expression of podocyte markers like nephrin has been shown before.^21^ We were able to confirm this effect using 2‐PM microscopy, where two phenotypes were visible: The first phenotype supports the hypothesis of podocyte loss, with less podocytes visible on the glomerular tuft. The second phenotype showed an unfused glomerulus, which might be a hint for developmental delay. It has been demonstrated previously that *bdnf* has beneficial effects on zebrafish kidney function and podocyte homoeostasis in a model of kidney injury. In this model, exogenous *bdnf* treatment rescued the expression of nephrin and almost completely restored podocyte morphology.^21^

Here, we show that *BDNF* mRNA may potentially serve as a new prognostic urinary biomarker for CKD. We also show that the expression of *BDNF* in human podocytes is locally restricted to the cell body and major processes. Furthermore, we give first insights into the involvement of *BDNF* in podocyte dedifferentiation and into its deregulation in DN. The KD of *bdnf* leads to impaired glomerular filtration as well as to podocyte loss and/or hindered migration of glomerular progenitors in an *in vivo* zebrafish model.

## CONFLICT OF INTEREST

The authors disclose no conflict of interests.

## AUTHOR CONTRIBUTIONS

N.E. and K.E. designed the study. S.S. and J.K. contributed to urine processing. J.K. performed urine mRNA PCR experiments. T.L. and P.K. contributed to statistical data analysis; N.E., U.Z., J.K., M.T.L., C.D.C. handled and analysed the biopsies. F.K. performed the (de‐)differentiation assay experiments. T.L., A.M.K. and F.S. performed zebrafish experiments. N.E., T.L. and K.E. analysed experimental data. T.L. did the figure design and literature search. N.E., T.L. and K.E. wrote the main manuscript text. All authors had approval of the final manuscript.

## References

[1] Auer PL , Teumer A , Schick U , et al. Rare and low‐frequency coding variants in CXCR2 and other genes are associated with hematological traits. Nat Genet. 2014;46:629‐634. 24777453 10.1038/ng.2962 PMC4050975

[2] Shungin D , Winkler TW , Croteau‐Chonka DC , et al. New genetic loci link adipose and insulin biology to body fat distribution. Nature. 2015;518:187‐196. 25673412 10.1038/nature14132 PMC4338562

[3] Grabe HJ , Assel H , Bahls T , et al. Cohort profile: Greifswald approach to individualized medicine (GANI_MED). J Transl Med. 2014;12:144. 24886498 10.1186/1479-5876-12-144 PMC4040487

[4] Zhang Q , Rothenbacher D . Prevalence of chronic kidney disease in population‐based studies: systematic review. BMC Public Health. 2008;8:117. 18405348 10.1186/1471-2458-8-117 PMC2377260

[5] Hallan SI , Vikse BE . Relationship between chronic kidney disease prevalence and end‐stage renal disease risk. Curr Opin Nephrol Hypertens. 2008;17:286‐291. 18408480 10.1097/MNH.0b013e3282f8b177

[6] Go AS , Chertow GM , Fan D , McCulloch CE , Hsu C . Chronic kidney disease and the risks of death, cardiovascular events, and hospitalization. N Engl J Med. 2004;351:1296‐1305. 15385656 10.1056/NEJMoa041031

[7] Canaud G , Bienaime F , Viau A , et al. AKT2 is essential to maintain podocyte viability and function during chronic kidney disease. Nat Med. 2013;19:1288‐1296. 24056770 10.1038/nm.3313

[8] Reiser J , Sever S . Podocyte biology and pathogenesis of kidney disease. Annu Rev Med. 2012;64:357‐366. 23190150 10.1146/annurev-med-050311-163340 PMC3736800

[9] Mundel P , Kriz W . Structure and function of podocytes: an update. Anat Embryol (Berl). 1995;192:385‐397. 8546330 10.1007/BF00240371

[10] Pavenstadt H , Kriz W , Kretzler M . Cell biology of the glomerular podocyte. Physiol Rev. 2003;83:253‐307. 12506131 10.1152/physrev.00020.2002

[11] Deller T , Mundel P , Frotscher M . Potential role of synaptopodin in spine motility by coupling actin to the spine apparatus. Hippocampus. 2000;10:569‐581. 11075827 10.1002/1098-1063(2000)10:5<569::AID-HIPO7>3.0.CO;2-M

[12] Putaala H , Soininen R , Kilpelainen P , Wartiovaara J , Tryggvason K . The murine nephrin gene is specifically expressed in kidney, brain and pancreas: inactivation of the gene leads to massive proteinuria and neonatal death. Hum Mol Genet. 2001;10:1‐8. 11136707 10.1093/hmg/10.1.1

[13] Numakawa T , Suzuki S , Kumamaru E , Adachi N , Richards M , Kunugi H . BDNF function and intracellular signaling in neurons. Histol Histopathol. 2010;25:237‐258. 20017110 10.14670/HH-25.237

[14] Ghosh A , Carnahan J , Greenberg ME . Requirement for BDNF in activity‐dependent survival of cortical neurons. Science. 1994;263:1618‐1623. 7907431 10.1126/science.7907431

[15] Horch HW , Katz LC . BDNF release from single cells elicits local dendritic growth in nearby neurons. Nat Neurosci. 2002;5:1177‐1184. 12368805 10.1038/nn927

[16] Sawai H , Clarke DB , Kittlerova P , Bray GM , Aguayo AJ . Brain‐derived neurotrophic factor and neurotrophin‐4/5 stimulate growth of axonal branches from regenerating retinal ganglion cells. J Neurosci. 1996;16:3887‐3894. 8656282 10.1523/JNEUROSCI.16-12-03887.1996 PMC6578616

[17] Lohof AM , Ip NY , Poo M . Potentiation of developing neuromuscular synapses by the neurotrophins NT‐3 and BDNF. Nature. 1993;363:350‐353. 8497318 10.1038/363350a0

[18] Ernfors P , Kucera J , Lee KF , Loring J , Jaenisch R . Studies on the physiological role of brain‐derived neurotrophic factor and neurotrophin‐3 in knockout mice. Int J Dev Biol. 1995;39:799‐807. 8645564

[19] Yamamoto M , Sobue G , Yamamoto K , Mitsuma T . Expression of mRNAs for neurotrophic factors (NGF, BDNF, NT‐3, and GDNF) and their receptors (p75 ngfr, TrkA, TrkB, and TrkC) in the adult human peripheral nervous system and nonneural tissues. Neurochem Res. 1996;21:929‐938. 8895847 10.1007/BF02532343

[20] Greene LA , Kaplan DR . Early events in neurotrophin signalling via Trk and p75 receptors. Curr Opin Neurobiol. 1995;5:579‐587. 8580709 10.1016/0959-4388(95)80062-x

[21] Li M , Armelloni S , Zennaro C , et al. BDNF repairs podocyte damage by microRNA‐mediated increase of actin polymerization. J Pathol. 2015;235:731‐744. 25408545 10.1002/path.4484 PMC4356648

[22] Waanders F , van Timmeren MM , Stegeman CA , Bakker SJL , van Goor H . Kidney injury molecule‐1 in renal disease. J Pathol. 2010;220:7‐16. 19921716 10.1002/path.2642

[23] Ichimura T , Hung CC , Yang SA , Stevens JL , Bonventre JV . Kidney injury molecule‐1: a tissue and urinary biomarker for nephrotoxicant‐induced renal injury. Am J Physiol Renal Physiol. 2004;286:F552‐F563. 14600030 10.1152/ajprenal.00285.2002

[24] Bonventre JV . Kidney Injury Molecule‐1 (KIM‐1): a specific and sensitive biomarker of kidney injury. Scand J Clin Lab Invest Suppl. 2008;241:78‐83. 18569971 10.1080/00365510802145059

[25] Zhang PL , Rothblum LI , Han WK , Blasick TM , Potdar S , Bonventre JV . Kidney injury molecule‐1 expression in transplant biopsies is a sensitive measure of cell injury. Kidney Int. 2008;73:608‐614. 18160964 10.1038/sj.ki.5002697 PMC2915578

[26] Zhao X , Zhang Y , Li L , et al. Glomerular expression of kidney injury molecule‐1 and podocytopenia in diabetic glomerulopathy. Am J Nephrol. 2011;34:268‐280. 21822010 10.1159/000330187 PMC3169370

[27] Yang L , Brooks CR , Xiao S , et al. KIM‐1‐mediated phagocytosis reduces acute injury to the kidney. J Clin Investig. 2015;125:1620‐1636. 25751064 10.1172/JCI75417 PMC4396492

[28] Siegerist F , Zhou W , Endlich K , Endlich N . 4D in vivo imaging of glomerular barrier function in a zebrafish podocyte injury model. Acta Physiol (Oxford, England). 2017;220:167‐173. 10.1111/apha.12754 27414464

[29] Schenk H , Müller‐Deile J , Kinast M , Schiffer M . Disease modeling in genetic kidney diseases: zebrafish. Cell Tissue Res. 2017;369:127‐141. 28331970 10.1007/s00441-017-2593-0

[30] Kotb AM , Müller T , Xie J , Anand‐Apte B , Endlich K , Endlich N . Simultaneous assessment of glomerular filtration and barrier function in live zebrafish. Am J Physiol Renal Physiol. 2014;307:F1427‐F1434. 25298528 10.1152/ajprenal.00029.2014 PMC4347739

[31] Drummond IA , Davidson AJ . Zebrafish kidney development. Methods Cell Biol. 2010;100:233‐260. 21111220 10.1016/B978-0-12-384892-5.00009-8

[32] Kramer‐Zucker AG , Wiessner S , Jensen AM , Drummond IA . Organization of the pronephric filtration apparatus in zebrafish requires Nephrin, Podocin and the FERM domain protein Mosaic eyes. Dev Biol. 2005;285:316‐329. 16102746 10.1016/j.ydbio.2005.06.038 PMC2836015

[33] Kotb AM , Simon O , Blumenthal A , et al. Knockdown of ApoL1 in Zebrafish larvae affects the glomerular filtration barrier and the expression of nephrin. PLoS ONE. 2016;11:e0153768. 27138898 10.1371/journal.pone.0153768 PMC4854397

[34] Siegerist F , Blumenthal A , Zhou W , Endlich K , Endlich N . Acute podocyte injury is not a stimulus for podocytes to migrate along the glomerular basement membrane in zebrafish larvae. Sci Rep. 2017;7:43655. 28252672 10.1038/srep43655 PMC5333633

[35] Hashimoto M , Heinrich G . Brain‐derived neurotrophic factor gene expression in the developing zebrafish. Int J Dev Neurosci. 1997;15:983‐997. 9641529 10.1016/s0736-5748(97)00017-8

[36] Kindt F , Hammer E , Kemnitz S , et al. A novel assay to assess the effect of pharmaceutical compounds on the differentiation of podocytes. Br J Pharmacol. 2017;174:163‐176. 27858997 10.1111/bph.13667 PMC5192948

[37] Bouter Y , Kacprowski T , Weissmann R , et al. Deciphering the molecular profile of plaques, memory decline and neuron loss in two mouse models for Alzheimer's disease by deep sequencing. Front Aging Neurosci. 2014;6:75. 24795628 10.3389/fnagi.2014.00075 PMC3997018

[38] Bollig F , Perner B , Besenbeck B , et al. A highly conserved retinoic acid responsive element controls wt1a expression in the zebrafish pronephros. Development (Cambridge, England). 2009;136:2883‐2892. 10.1242/dev.031773 19666820

[39] Müller T , Rumpel E , Hradetzky S , et al. Non‐muscle myosin IIA is required for the development of the zebrafish glomerulus. Kidney Int. 2011;80:1055‐1063. 21849970 10.1038/ki.2011.256

[40] Endlich N , Simon O , Göpferich A , et al. Two‐photon microscopy reveals stationary podocytes in living zebrafish larvae. J Am Soc Nephrol. 2014;25:681‐686. 24309184 10.1681/ASN.2013020178 PMC3968489

[41] Foley RN , Wang C , Snyder JJ , Collins AJ . Cystatin C levels in U.S. adults, 1988‐1994 versus 1999‐2002: NHANES. Clin J Am Soc Nephrol. 2009;4:965‐972. 19339409 10.2215/CJN.05281008 PMC2676178

[42] Peralta CA , Whooley MA , Ix JH , Shlipak MG . Kidney function and systolic blood pressure new insights from cystatin C: data from the Heart and Soul Study. Am J Hypertens. 2006;19:939‐946. 16942937 10.1016/j.amjhyper.2006.02.007 PMC2771570

[43] Jafar TH , Chaturvedi N , Hatcher J , Levey AS . Use of albumin creatinine ratio and urine albumin concentration as a screening test for albuminuria in an Indo‐Asian population. Nephrol Dial Transplant. 2007;22:2194‐2200. 17405790 10.1093/ndt/gfm114

[44] Rifkin DE , Katz R , Chonchol M , et al. Albuminuria, impaired kidney function and cardiovascular outcomes or mortality in the elderly. Nephrol Dial Transplant. 2010;25:1560‐1567. 20008829 10.1093/ndt/gfp646 PMC3307251

[45] Jeon YK , Kim MR , Huh JE , et al. Cystatin C as an early biomarker of nephropathy in patients with type 2 diabetes. J Korean Med Sci. 2011;26:258‐263. 21286018 10.3346/jkms.2011.26.2.258 PMC3031011

[46] Wickman L , Afshinnia F , Wang SQ , et al. Urine podocyte mRNAs, proteinuria, and progression in human glomerular diseases. J Am Soc Nephrol. 2013;24:2081‐2095. 24052633 10.1681/ASN.2013020173 PMC3839551

[47] Fukuda A , Wickman LT , Venkatareddy MP , et al. Urine podocin:nephrin mRNA ratio (PNR) as a podocyte stress biomarker. Nephrol Dial Transplant. 2012;27:4079‐4087. 22863839 10.1093/ndt/gfs313 PMC3494841

[48] Wang G , Lai FM , Lai K , Chow K , Li KP , Szeto C . Messenger RNA expression of podocyte‐associated molecules in the urinary sediment of patients with diabetic nephropathy. Nephron Clin Pract. 2007;106:c169‐c179. 17596726 10.1159/000104428

[49] Ju W , Smith S , Kretzler M . Genomic biomarkers for chronic kidney disease. Transl Res. 2012;159:290‐302. 22424432 10.1016/j.trsl.2012.01.020 PMC3329158

[50] Han WK , Bailly V , Abichandani R , Thadhani R , Bonventre JV . Kidney Injury Molecule‐1 (KIM‐1): a novel biomarker for human renal proximal tubule injury. Kidney Int. 2002;62:237‐244. 12081583 10.1046/j.1523-1755.2002.00433.x

[51] Yin C , Wang N . Kidney injury molecule‐1 in kidney disease. Ren Fail. 2016;38:1567‐1573. 27758121 10.1080/0886022X.2016.1193816

[52] Vaidya VS , Ozer JS , Dieterle F , et al. Kidney injury molecule‐1 outperforms traditional biomarkers of kidney injury in preclinical biomarker qualification studies. Nat Biotechnol. 2010;28:478‐485. 20458318 10.1038/nbt.1623 PMC2885849

[53] Carrero JJ . Gender differences in chronic kidney disease: underpinnings and therapeutic implications. Kidney Blood Press Res. 2010;33:383‐392. 20948227 10.1159/000320389

[54] Si H , Banga RS , Kapitsinou P , et al. Human and murine kidneys show gender‐ and species‐specific gene expression differences in response to injury. PLoS ONE. 2009;4:e4802. 19277126 10.1371/journal.pone.0004802 PMC2652077

[55] Hodgin JB , Bitzer M , Wickman L , et al. Glomerular aging and focal global glomerulosclerosis: a podometric perspective. J Am Soc Nephrol. 2015;26:3162‐3178. 26038526 10.1681/ASN.2014080752 PMC4657829

[56] Naik AS , Afshinnia F , Cibrik D , et al. Quantitative podocyte parameters predict human native kidney and allograft half‐lives. JCI Insight. 2016;1:e86943. 27280173 10.1172/jci.insight.86943 PMC4894348

[57] Brooks CR , Yeung MY , Brooks YS , et al. KIM‐1‐/TIM‐1‐mediated phagocytosis links ATG5‐/ULK1‐dependent clearance of apoptotic cells to antigen presentation. EMBO J. 2015;34:2441‐2464. 26282792 10.15252/embj.201489838 PMC4601664

[58] Nikoletopoulou V , Sidiropoulou K , Kallergi E , Dalezios Y , Tavernarakis N . Modulation of autophagy by BDNF underlies synaptic plasticity. Cell Metab. 2017;26:230‐242.e5. 28683289 10.1016/j.cmet.2017.06.005

[59] Kononenko NL , Claßen GA , Kuijpers M , et al. Retrograde transport of TrkB‐containing autophagosomes via the adaptor AP‐2 mediates neuronal complexity and prevents neurodegeneration. Nat Commun. 2017;8:14819. 28387218 10.1038/ncomms14819 PMC5385568

