# Supplementary figures and images for: BDNF: mRNA expression in urine cells of patients with chronic kidney disease and its role in kidney function
Source: J Cell Mol Med. 2018 Aug 21;22(11):5265–77. doi: 10.1111/jcmm.13762 (PMC6201371; doi:10.1111/jcmm.13762)

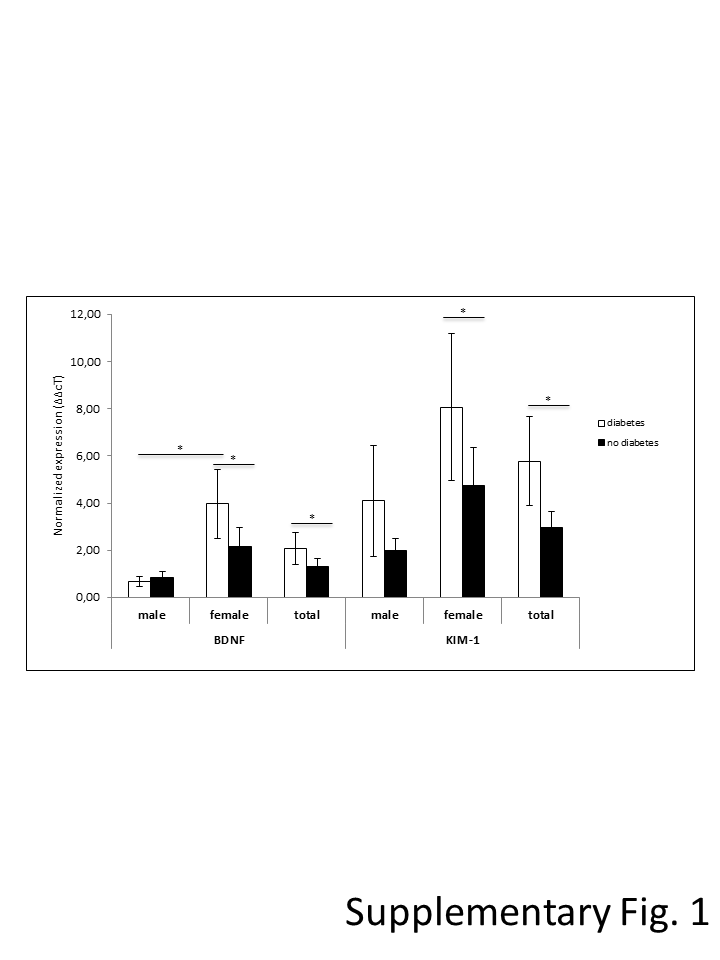

Supplement: Supplementary file 1 [file JCMM-22-5265-s001.TIF]

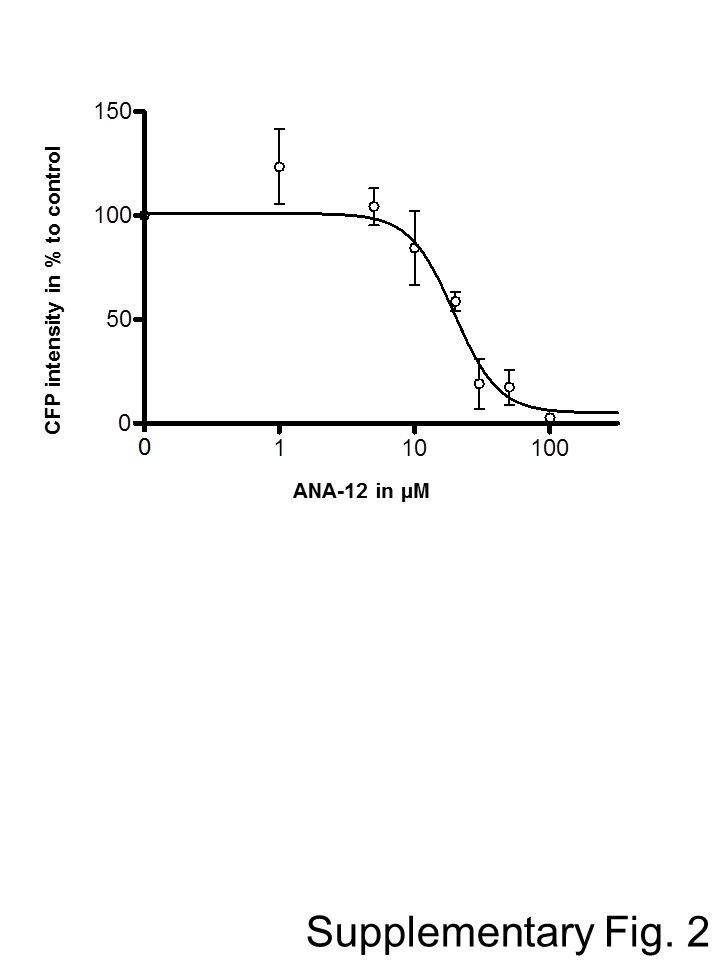

Supplement: Supplementary file 2 [file JCMM-22-5265-s002.TIF]

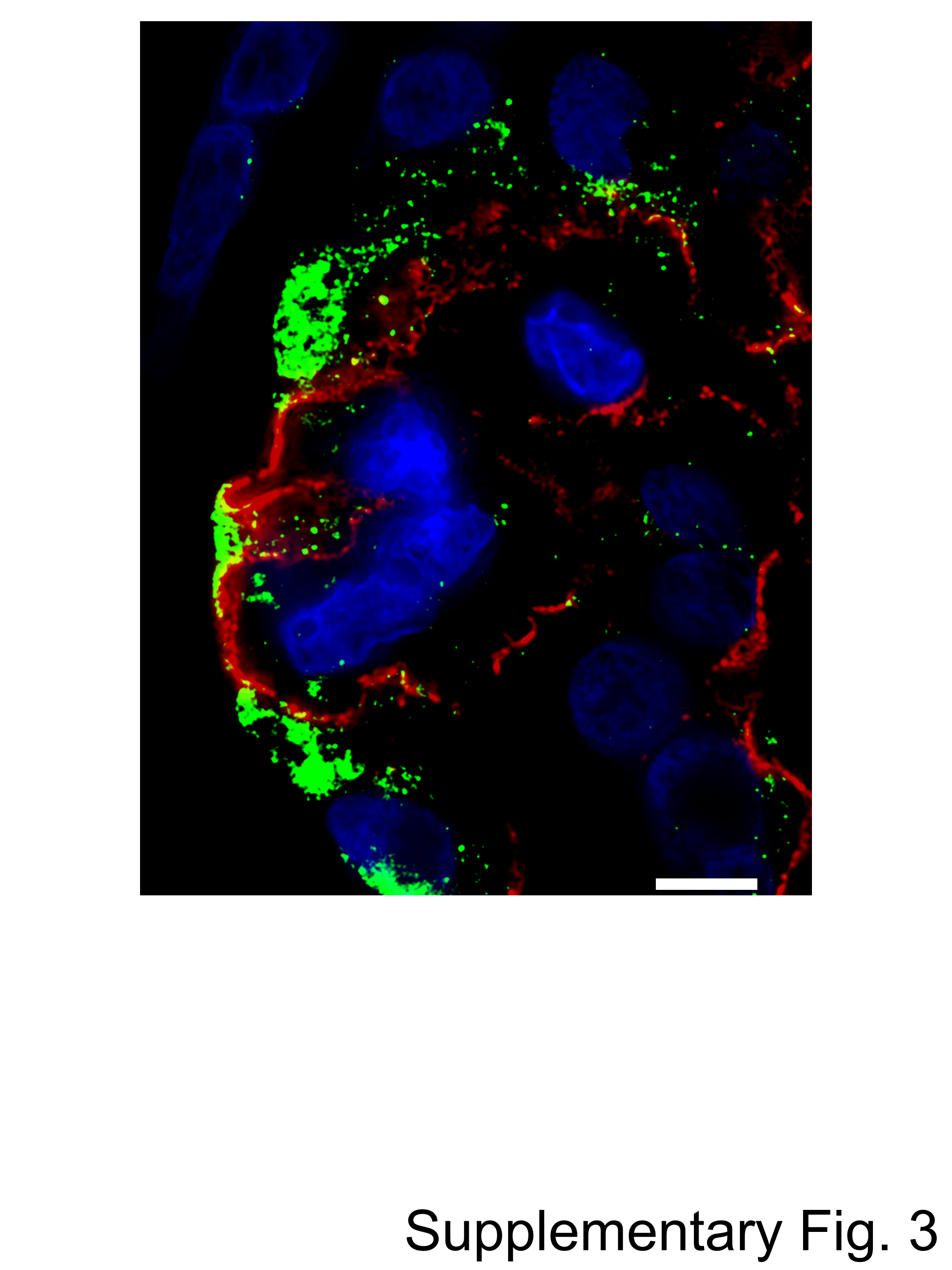

Supplement: Supplementary file 3 [file JCMM-22-5265-s003.TIF]

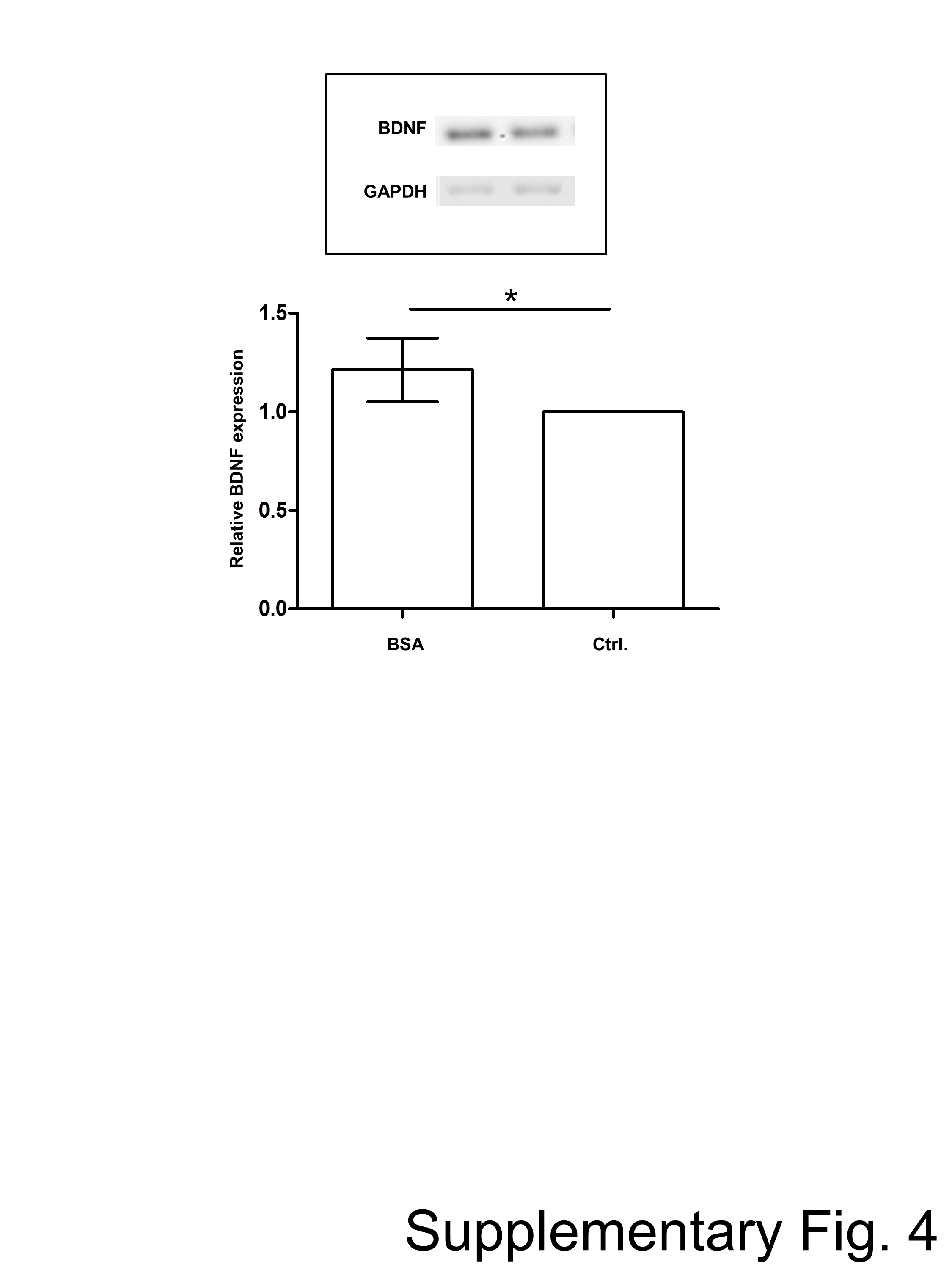

Supplement: Supplementary file 4 [file JCMM-22-5265-s004.TIF]
