# Supplementary material for: BDNF: mRNA expression in urine cells of patients with chronic kidney disease and its role in kidney function
Source: J Cell Mol Med. 2018 Aug 21;22(11):5265–77. doi: 10.1111/jcmm.13762 (PMC6201371; doi:10.1111/jcmm.13762)
Supplement: Supplementary file 9 [file JCMM-22-5265-s009.docx]

**Supplementary Methods**

**Urine RNA preparation and quantitative RT-PCR assay**

The total urine pellet RNA was isolated using the protocol of the RNeasy Plus Universal Kit (Qiagen, Hilden, Germany). The cDNA was synthesized using the Superscript II reverse transcriptase and oligo(dT)_12-18_ primer (Thermo Fisher Scientific, Waltham, MA, USA) according to the manufacturer`s instructions. Quantification of relative *NPHS1*, *NPHS2*, *PODXL*, *KIM-1*, *BDNF* and *CTSL* mRNA abundances was performed with the Light Cycler^®^ Nano instrument using FastStart Essentials DNA Probes Master and Universal Probe Library probes (Roche Applied Biosystem, Mannheim, Germany). Two independent cDNA reverse transcriptions from the same RNA sample were performed, each measured in duplicate. Relative expression was normalized to *GAPDH* and total kidney cDNA as inter-run-calibrator. Analyses were performed with the Light Cycler^®^ Nano SW 1.0 software (Roche Applied Biosystems) using the ΔΔCt-method.

**Histology and immunofluorescence**

All histology was performed on 3.7% FFPE human kidney tissue. 4-µm sections of kidney samples were deparaffinized and rehydrated in a routine manner. Sections were incubated for 5 minutes at 95°C with 10 mM citrate buffer (pH 6) for antigen unmasking followed by blocking (2% FBS, 2% bovine serum fraction V, 0.2% fish gelatine, and PBS) for 1 hour. The following antibodies were used: rabbit anti-*BDNF* (1:25, Santa Cruz Biotechnology, Santa Cruz, CA, USA), rabbit anti-*KIM-1* (1:25, LSBio, Seattle, WA, USA), mouse anti-synaptopodin (1:10, Progen, Heidelberg, Germany) and guinea-pig anti-nephrin (1:100 Progen). Primary antibodies were applied overnight at 4°C. After washing three times with PBS, Cy2- and Cy3-labeled secondary antibodies (1:300, Dianova, Hamburg, Germany) were applied for 1 hour at RT. Nuclei were counterstained with 1 mg/100 mL Hoechst 33342 (Sigma-Aldrich, Darmstadt, Germany). Sections were mounted in Mowiol (Carl Roth, Karlsruhe, Germany) and documented using a Leica TCS SP5 confocal laser scanning microscope (Leica Microsystems, Wetzlar, Germany). 30-µm paraffin sections were permeabilized with 0.3% Triton X-100 (Merck, Darmstadt, Germany) for 2 minutes after blocking. Three-dimensional image reconstructions were created with the Volocity software 6.3 (Improvision, Coventry, UK).

**Zebrafish RNA isolation and (q)RT-PCR**

For RNA isolation, approximately 15 zebrafish larvae per group were treated with 900 µL Tri-Reagent (Sigma-Aldrich) and were homogenized by addition of ceramic beads and serial vortexing for 5 min. RNA isolation was performed according to the manufacturer’s protocol. Reverse transcriptase reaction was performed with the QuantiTect Reverse Transcription Kit (Qiagen) according to manufacturer’s instructions. Controls without template and without reverse transcriptase were included. The RT-PCR reaction was performed for the target genes *bdnf*, *nphs2* and the reference gene *zgc:158463* using DreamTaq Green DNA Polymerase and Master Mix (Thermo Fisher Scientific). qRT-PCRs for *nphs2* were performed on a Bio-Rad iCycler Thermal Cycler with the iQ5 Multicolor Real-Time PCR Detection System (Bio-Rad, Hercules, CA, USA) using the iQ SYBR Green Supermix (Bio-Rad) according to manufacturer’s instructions. Additionally, e*ef1a1l1* was used as a second reference gene. Data were analyzed using the Bio-Rad iQ5 2.1 software with the ΔΔCt method and results are given as normalized fold expression compared to the CtrlMO treated group.

**Zebrafish immunohistology**

The following antibodies were used: anti-zebrafish nephrin (1:2000; Innovagen, Sweden). As secondary antibody, Cy3-conjugated anti-rabbit antibody (1:200; Dianova) was applied. Alexa Fluor 546-conjugated phalloidin (Thermo Fisher Scientific) and Hoechst 33342 (0.013 mg/mL; Sigma-Aldrich) were used to visualize F-actin and nuclei, respectively.

**Microarray analysis**

Human kidney biopsies for Affymetrix microarray expression data were obtained within the scope of the European Renal cDNA Bank Kröner-Fresenius Biopsy Bank. [44,45] Biopsies were obtained from patients after an informed written consent, approved by the local ethics committees. Isolation of glomeruli, total RNA isolation, reverse transcription and linear amplification were performed as described previously. [46] Microarray expression data was obtained from individual DN patients. Pre-transplantation kidney biopsies from living donors were used as controls. Fragmentation, hybridization, staining, and imaging were performed with the Affymetrix HG-U 133plus 2.0 system (Affymetrix, Santa Clara, CA), according to the manufacturer’s instructions. CEL file normalization was performed with the Robust Multichip Average method using RMAExpress (Version 1.0.5) and the human Entrez-Gene custom CDF annotation from Brain Array version 18 (http://brainarray.mbni.med.umich.edu/Brainarray/default.asp). To identify differentially expressed genes, the SAM (Significance analysis of Microarrays) method was applied using TiGR (MeV, Version 4.8.1). [47] Q-values ≤ 5% were considered statistically significant.

**Structured illumination microscopy**

2D structured illumination microscopy was performed as previously described [61] on a Zeiss Elyra SP.1 system (Zeiss Microscopy, Jena, Germany) equipped with a 63x (NA 1.4) oil immersion objective and the Zeiss ZEN software .

**Protein-overload experiments**

We used cultured murine podocytes that have been described previously [62]. Podocytes were kept at 38°C for 3 weeks before use for differentiation. One hour before BSA treatment, podocytes cell culture medium was changed and supplemented with 2 % FBS. BSA (Sigma-Aldrich, cell-culture grade) was diluted in cell culture medium (2 % FBS) at 30 mg/mL. Podocytes were treated for 20 h and analysed by RT-PCR for BDNF and GAPDH.
