# Supplementary material for: BDNF: mRNA expression in urine cells of patients with chronic kidney disease and its role in kidney function
Source: J Cell Mol Med. 2018 Aug 21;22(11):5265–77. doi: 10.1111/jcmm.13762 (PMC6201371; doi:10.1111/jcmm.13762)
Supplement: Supplementary file 10 [file JCMM-22-5265-s010.docx]

**Supplementary Figure 1.**

Urine sediment *BDNF* and *KIM-1* mRNA expression in diabetics and non-diabetics. *BDNF* and *KIM-1* mRNA levels were significantly higher in urine sediments from CKD patients with diabetes than from non-diabetics. When analyzed according to gender, this effect was seen in female but not in male CKD patients. [* p<0.05; n.s.=not significant; error bars=SEM]

**Supplementary Figure 2.**

TrkB antagonism affects podocyte de-/differentiation. The figure shows the results of the podocyte de-/differentiation assay. CFP fluorescence intensity in relation to control-treated glomeruli is shown. TrkB-receptor inhibitor ANA-12 was applied in concentrations from 1-100 µM. TrkB-receptor antagonism led to concentration-dependent enhanced podocyte dedifferentiation, after 6 days with an IC-50 of 19.56 µM.

**Supplementary Figure 3.**

BDNF is expressed specifically in podocytes. A normal human kidney section was co-stained for BDNF and the slit diaphragm protein nephrin. Imaging was performed by the super-resolution microscopic technique SIM (Structured Illumination Microscopy). BDNF is expressed in the cell body and major processes of podocytes, whereas nephrin is detected in the foot processes. [Scale bar=5 µm]

**Supplementary Figure 4.**

Protein-overload leads to BDNF upregulation. Cultured murine podocytes were treated with 30 mg/mL BSA for 20 h. The figure shows RT-PCR results for BDNF and GAPDH as normalization. BDNF expression is slightly (1.2-fold) upregulated in BSA treated podocytes in comparison to controls. [* p<0.05; n=3, error bars=SD]

**Supplementary Movie 1.**

2-PM 3D reconstruction of a larval ET zebrafish glomerulus (4 dpf) treated with bdnf morpholinos.

**Supplementary Movie 2.**

2-PM z-stack of a larval ET zebrafish glomerulus (4 dpf) treated with bdnf morpholinos.

**Supplementary Movie 3.**

2-PM 3D reconstruction of a larval ET zebrafish glomerulus (4 dpf) treated with control morpholinos.

**Supplementary Movie 4.**

2-PM z-stack of a larval ET zebrafish glomerulus (4 dpf) treated with control morpholinos.
